# Clinical and Paraclinical Features in Pregnancies Associated With Renal Impairment Due to Hypertensive Complications

**DOI:** 10.7759/cureus.57849

**Published:** 2024-04-08

**Authors:** Daniela-Catalina Meca, Monica Mihaela Cirstoiu

**Affiliations:** 1 Department of Obstetrics and Gynecology, University Emergency Hospital in Bucharest, "Carol Davila" University of Medicine and Pharmacy, Bucharest, ROU

**Keywords:** stillbirth, preterm birth, low birth weight, acute renal failure, neonatal outcome, pregnancy

## Abstract

Background

An association between renal impairment and hypertensive complications occurring during pregnancy has been shown in a limited number of studies. As a consequence of a lack of clear criteria for diagnostic certainty, acute renal failure during pregnancy is a challenging pathology to diagnose, mainly due to the physiological reduction of nitrogen retention parameters. In light of the fact that renal injury is associated with a poor maternal and fetal prognosis, this study aims to determine the maternal demographic features and the cut-off of serum creatinine that can lead to a heightened risk of prematurity, stillbirth, intrauterine growth restriction, or the necessity of neonatal intensive care.

Methods

We performed a study that included a cohort of 45 pregnant women with acute renal injury who delivered in the Department of Obstetrics and Gynecology of the University Emergency Hospital in Bucharest between January 1, 2017, and December 31, 2022, a cohort of 45 pregnant women with a value of serum creatinine between 0.8 and 1 mg/dL, and a cohort of 45 pregnant women, selected at random, with a value of serum creatinine under 0.8 mg/dL, who delivered in the same period in the aforementioned unit. The analysis included neonatal outcomes (preterm birth, intrauterine growth restriction, stillbirth, Apgar score calculated at one minute, the need for neonatal intensive care), maternal demographic features, medical and obstetrical history, and paraclinical parameters.

Results

The incidence of acute renal injury was 0.33% for the entire cohort of patients who gave birth in our hospital. Out of that lot, 65.21% of the cases of acute renal impairment associated with pregnancy were caused by hypertensive complications. The mean age of patients with acute kidney injury (AKI) was 29.4 ± 6.66, preponderantly primiparous. The majority of the neonates from patients with AKI (62.22%) were born with a birth weight under 2.500 grams. Preterm deliveries were preponderant (66.66%) in patients with AKI, while in the control group, the incidence of preterm deliveries was 48.88%. Stillbirth in the AKI group had an incidence of 13.33%, while in the control group, there were none. Due to these neonatal complications, most of the newborns in the AKI group needed neonatal intensive care. An important percentage of the patients who developed AKI (40%) did not benefit from proper medical care during pregnancy or before admission to our unit. The cutoff of 1.09 mg/dL of serum creatinine level was established following receiver operating characteristic curve analysis.

Conclusion

AKI during pregnancy is associated with hypertensive disorders, low birth weight, and preterm deliveries.

## Introduction

Pregnancy-related acute kidney injury (AKI) represents a serious health problem. Its incidence has been decreasing due to improvements in reproductive health care; however, in low- and middle-income countries, pregnancy-related AKI is the most common reason for dialysis [1-7].

It is generally accepted that hypertensive disorders of pregnancy, especially preeclampsia with severe characteristics and hemolysis, elevated liver enzymes, and low platelets (HELLP) syndrome, are the main causes of AKI [1,6,8-11]. The definition of renal insufficiency in the setting of hypertension disorders during pregnancy, according to the American College of Obstetricians and Gynecologists, is a serum creatinine level >1.1 mg/dL or doubling it in the absence of renal disease [11,12].

Usually, AKI occurs during pregnancy in the second trimester; however, this does not exclude the possibility of it appearing in any trimester or the postpartum period [11]. It must be highlighted that this pathology is associated with increased rates of maternal mortality and fetal loss, varying from 30% to 60% [11,13]. Between 1998 and 2009, in the United States of America, more than 17% of deaths during delivery hospitalization and more than 31% of deaths during postpartum hospitalization occurred among patients with acute renal failure of any etiology [11,14].

Regarding fetal and maternal outcomes, a 2017 systemic review and meta-analysis showed that pregnant patients with AKI, compared to those without AKI, had a higher incidence of maternal complications: cesarean delivery, obstetrical hemorrhage, placental abruption, disseminated intravascular coagulation, longer need for intensive care, and neonatal complications: stillbirth or perinatal death, lower mean gestational age at delivery, or lower birth weight [7,11].

Regarding the treatment of AKI in pregnancy, the most important step is to determine the underlying pathology and to decide on a personalized management strategy that includes the moment of delivery [2]. It is crucial to follow up with patients with an episode of pregnancy-related AKI, especially those with the etiology of hypertension disorders during pregnancy [2]. In most cases, women who had proteinuria due to preeclampsia at 6 months after delivery will show no sign of it [2,15]. Hence, if a patient has persistent proteinuria due to preeclampsia in the postpartum period, it is necessary to consult a nephrologist; studies showed that most of the women with this modification developed subsequent renal disease [2,16]. Regarding the long-term consequences of pregnancy-related AKI, it was commonly thought that renal dysfunction reversed after delivery in cases of preeclampsia and HELLP syndrome; nowadays, there is proof that in these cases, AKI leads to chronic future renal disease and cardiovascular damage [11].

## Materials and methods

We performed a descriptive, retrospective study that included 45 pregnant women with acute renal failure caused by hypertensive complications who delivered in the Department of Obstetrics and Gynecology of the University Emergency Hospital in Bucharest between January 1, 2017, and December 31, 2022, a borderline value serum creatinine group of 45 patients, and a control group of 45 randomized pregnant women with normal values of serum creatinine who delivered in the same period in the same unit. The data regarding maternal and fetal parameters were collected from the database system of the University Emergency Hospital in Bucharest and the hospitalization sheets. The patients included in the study signed the informed consent form at admission.

We included all the cases of pregnant women with acute renal failure caused by hypertensive complications: severe preeclampsia, eclampsia, and placental abruption. The borderline serum creatinine group (0.8-1 mg/dL) was established due to the lack of diagnostic criteria for AKI and the physiological reduction in serum creatinine levels during normal pregnancies. The exclusion criteria were as follows: age under 18 years, the absence of informed consent, and the presence of renal failure caused by sepsis or hemorrhage.

The study was approved by the Ethical Committee of the University Emergency Hospital in Bucharest (approval number: 26407/17.05.2021). Data analysis was performed using SPSS Statistics version 26.0 (IBM Corp. Released 2019. IBM SPSS Statistics for Windows, Version 26.0. Armonk, NY: IBM Corp.), and the results that were considered statistically significant had p<0.05. The chi-square test was performed to establish the interdependence of nominal variables, and the Pearson correlation test was used to underline the impact of serum creatinine level on fetal parameters (gestational age at delivery, Apgar score, fetal weight).

## Results

During our six years of study, out of 13,723 births registered in the University Emergency Hospital in Bucharest, only 45 patients developed acute renal failure caused by hypertensive complications during pregnancy and were eligible for our study. Over this period of time, the incidence of acute renal injury associated with hypertensive complications was 0.33%.

All three groups in the study consisted of an identical number of cases, with the exception that the borderline and the control group cases were selected randomly. All pregnancies were singletons, and none of the patients in the AKI group underwent assisted reproduction technology.

The cutoff of 1.09 mg/dL of serum creatinine level was established using receiver operating characteristic curve analysis based on fetal weight and one-minute Apgar score. The clinical and paraclinical features are described in Table 1.

**Table 1:** Demographic description of the groups under study BMI: body mass index, HELLP: hemolysis, elevated liver enzymes, and low platelets, eGFR: estimated glomerular filtration rate, NICU: neonatal intensive care unit

Demographic data		Acute renal failure group	Borderline group	Control group	p-value
Maternal data					
Maternal age, years (mean ± SD)		29.4 ± 6.66	31.68 ± 5.38	31.66 ± 5.38	0.980
BMI (mean ± SD)		24.97 ± 4.38	24.20 ±4.54	23.67 ± 4.96	0.230
Parity	Primiparous N(%)	32 (71.11%)	31 (68.89%)	32 (71.11%)	0.024
	Multiparous N(%)	13 (28.89%)	14 (31.11%)	13 (28.89%)	
Gestational diabetes N(%)		6 (13.33%)	2 (4.44%)	9 (20%)	0.011
Thrombophilia N(%)		3 (6.67%)	6 (13.33%)	20 (44.44%)	0.083
Hypertensive complications	Preeclampsia N(%)	25 (55.56%)	39 (86.67%)	36 (80%)	0.020
	Eclampsia N(%)	7 (15.55%)	1 (2.22%)	0	
	HELLP syndrome N(%)	4 (8.89%)	2 (4.44%)	4 (8.89%)	
	Placental abruption N(%)	9 (20%)	3 (6.67%)	5 (11.11%)	
Investigated pregnancy N(%)		27 (60%)	40 (88.89%)	38 (84.44%)	0.003
Urinary tract infection N(%)		9 (20%)	6 (13.33%)	7 (15.56%)	0.083
Days of hospitalization (mean ± SD)		10 ± 7.02	7.11 ±6.25	7.6 ± 6.11	0.184
Serum creatinine at admission, mg/dL (mean ± SD)		1.59 ± 0.80	0.82 ± 0.03	0.60 ± 0.10	< .001>
Serum creatinine at discharge (mean ± SD)		1.29 ± 0.85	0.69± 0.10	0.60 ± 0.11	0.217
Serum urea at admission (mean ± SD)		44.40 ± 19	26.72 ± 9.63	22.02 ± 7.12	0.285
Serum urea at discharge (mean ± SD)		46.27 ± 23.07	28.45 ± 10.32	22.30 ± 7.87	0.856
Proteinuria, mg/L (mean ± SD)		275.55 ± 93.41	244.77 ± 93.41	217.55 ± 108.96	0.584
eGFR, mL/min/1.73 m^2 ^(mean ± SD)		55.67 ± 20.39	95.95 ± 11.01	127.78 ± 46.94	0.034
Neonatal outcomes					
Gestational age (weeks) (mean ± SD)		34.37 ± 3.90	36.35 ± 3.74	35 ± 3.71	0.892
Birth weight (grams) (mean ± SD)		2226.33 ± 774.25	2653.11 ± 971.81	2551.33 ± 939.19	< .001>
Preterm birth N(%)		30 (66.67%)	15 (33.33%)	22 (48.89%)	0.522
Intrauterine growth restriction N(%)		24 (53.33%)	21 (46.67%)	18 (40%)	0.037
Stillbirth N(%)		6 (13.33%)	2 (4.44%)	0	0.22
1 min Apgar score (mean ± SD)		5.42 ± 3.30	7.55 ± 2.46	7.35 ± 2.14	0.002
NICU admission N(%)		25 (64.10%)	14 (32.55%)	20 (44.44%)	0.146

We found that the p-value is statistically significant as we talk about the parity or the presence of gestational diabetes or hypertensive complications in the groups under study. The fact that the patients in the control group had proper regular check-ups and medical assistance during pregnancy made an important difference when compared to the other groups of study.

**Figure 1:**
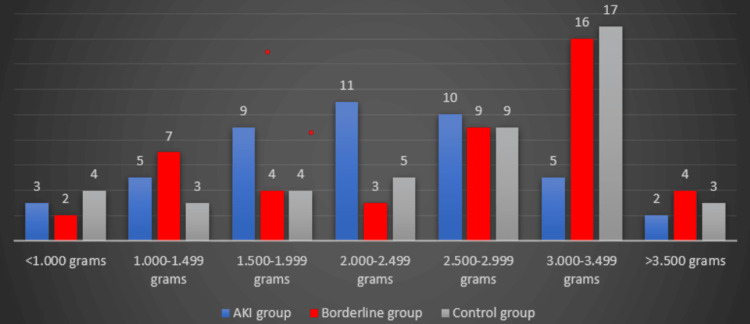
Distribution of birth weight in the three analyzed groups

Regarding the laboratory analysis, the serum creatinine at admission was found to be statistically significant between groups. As for the newborns’ features, the birth weight difference between the three groups is statistically significant. Figure 1 represents the distribution of birth weight in the studied groups. It shows that most newborns (62.22%) from patients with AKI had a weight of less than 2,500 grams, while in the borderline group, only 35.55% of newborns have a registered weight under 2,500 grams. The majority of newborns (64.44%) delivered by the women in the control group had a weight above 2,500 grams.

**Figure 2:**
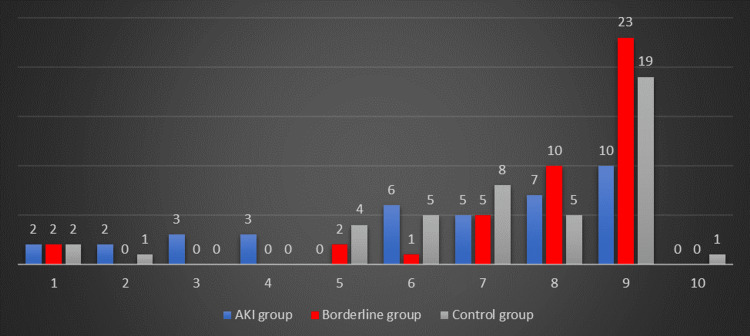
Distribution of one-minute Apgar score in the three analyzed groups

The one-minute Apgar score was also found to be statistically significant; therefore, in the AKI group, the mean one-minute Apgar score was 5.42, while in the control group, it was 7.35. Figure 2 illustrates the distribution of the one-minute Apgar score in the groups studied.

**Figure 3:**
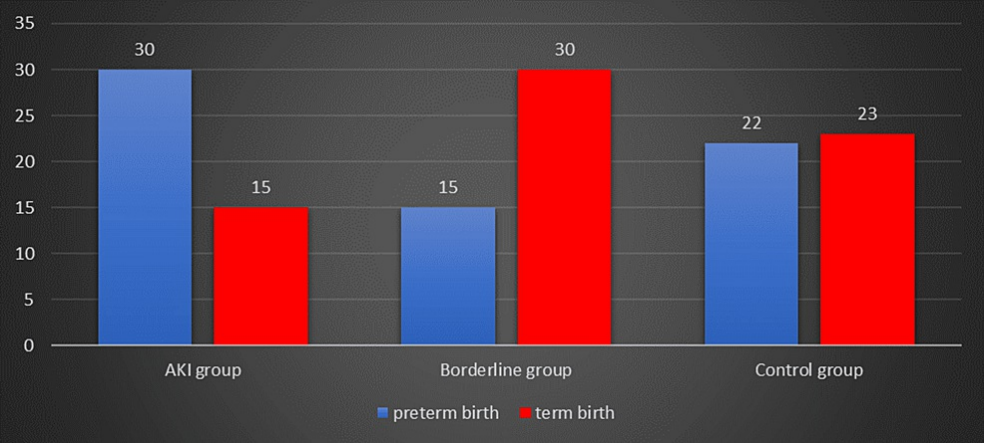
Distribution of preterm birth in the three analyzed groups

Preterm births were found to have no statistical significance, although the distribution of preterm newborns (Figure 3) revealed that most newborns (66.67%) of patients with AKI were delivered preterm. Preterm birth incidence in the borderline group was 33.33%, whereas in the control group it was 48.89%.

The AKI group displayed an incidence of stillbirth of 13.33%, whereas the control group had none. Moreover, the majority of newborns (64.10%) delivered by patients with AKI needed neonatal intensive care. The mode of delivery was the emergency cesarean section in 100% of all three groups, justified by hypertensive complications, imminent rupture of the uterus, or acute fetal distress.

## Discussion

Pregnancy-related acute kidney injury can occur during the antenatal, intrapartum, or postpartum period, and it can be a life-threatening complication for both the mother and the child [17].

Our study showed an incidence of acute renal injury associated with hypertensive disorders of pregnancy of 65.21%, taking into consideration that of a total of 13,732 births that occurred in our hospital, only 69 patients developed AKI. The study’s findings support those of other studies, which have shown that, worldwide, hypertensive complications during pregnancy are the most common cause of pregnancy-related AKI [1,6,8-11]. In a study that included 150 pregnant women with AKI, the most common causes of AKI were hypertension disorders of pregnancy with an incidence of 48%, puerperal sepsis at 45%, and hemorrhage at 34% [17]. In a systemic review and meta-analysis, patients with hypertensive disorders during pregnancy who developed AKI represented 36.6% [18].

In this paper, the mean age of patients with AKI was 29.4 ± 6.6 years, while in the borderline group, the mean age was 31.68 ± 5.38 years, and in the control group, it was 31.66 ± 5.38 years. Regarding the patients with pregnancy-related AKI, other studies reported a mean age of 26.65 ± 3.18, 24.16 ± 5.02, and 25.00 ± 00 years [17,19,20].

We found that most newborns (62.22%) of patients with AKI had a birth weight below 2.500 grams. Extremely preterm birth (under 28 weeks of pregnancy) was found in 8.89% (four cases). Very preterm birth (28 to less than 32 weeks of pregnancy) was found in 11.11% of the cases (five cases), while moderate to late preterm (32-37 weeks of pregnancy) was encountered in 23 cases (51.11%). Therefore, the mean weight of the newborns from patients with AKI and those from the control group was significantly different (2.226 grams vs. 2.551 grams). These findings are consistent with those of other studies that showed an incidence of 82.2% of neonates under 2500 grams from pregnancies with AKI or a fetal weight difference between pregnant women with or without AKI (3.258 grams vs. 3.370 grams) [17,21]. A reason for the high incidence of cases of low birth weight can be due to the high incidence of intrauterine growth restriction in the AKI group (53.33%).

Preterm births were recorded in 66.67% of the patients in the AKI group, while in the control group, the incidence was 48.89%. The percentage of preterm newborns in the present study is higher compared to various other studies, which reported an incidence of 28.5%, 53%, and 40.9%, respectively [17,18,22,23]. The explanation of preterm labor and birth is highly possible because of the stimulation of the center of uterine contraction by the excess of carbon dioxide in the blood caused by metabolic acidosis [18]. Due to the significant number of preterm deliveries, the majority of newborns (64.10%) from patients with AKI needed neonatal intensive care.

We found an incidence of 13.33% of stillbirths in the AKI groups, while in the control group, there were none. Other studies found higher incidences of stillbirths, at 38.4% and 25.4%, respectively [18,19]. In our study, the one-minute Apgar score was lower in the AKI group compared with the value of the newborns in the other two groups, but this can be explained by the fact that most patients with AKI had a preterm birth.

Study limitations

The main limitation of this study is the small sample size, attributable to the rarity of pregnancy-related AKI. Another limitation of the study is that a large number of the patients who developed AKI did not undergo adequate medical visits; therefore, we cannot have a suitable history of the patient and disease.

## Conclusions

Pregnancy-related AKI represents a serious health problem with serious maternal and neonatal consequences. AKI during pregnancy is associated with low birth weight, preterm deliveries, and stillbirth. As a consequence of these important neonatal complications, the majority of the babies delivered by patients with AKI needed neonatal intensive care. According to our study, 40% of patients who developed AKI did not receive proper medical care during pregnancy. A better program for specific investigations to prevent, diagnose, and manage AKI during pregnancy may reduce maternal and fetal complications.
